# Management of Duodenal Atresia in the setting of Congenital Leukemia with Massive Hepatomegaly

**Published:** 2013-07-01

**Authors:** Sathyaprasad C Burjonrappa

**Affiliations:** Attending Pediatric Surgeon, Children's Hospital of NJ and St.Barnabas Medical Center and Clinical Assistant Professor, Dept of Pediatric Surgery University of Buffalo

**Keywords:** Down's syndrome, Duodenal atresia, Transient myeloproliferative disorder

## Abstract

Down's syndrome (DS) is associated with duodenal atresia (DA) in about 8-10% of cases. Transient Myeloproliferative Disorder (TMD)/Acute Myeloid Leukemia (AML) is also associated with the trisomy 21 mutation. The occurrence of the two conditions together complicates the diagnosis and surgical management of the DA. We discuss the technical aspects of management of the DA in this clinical setting.

## INTRODUCTION

There is about 40% incidence of DA in the setting of DS [1]. DS is associated with a plethora of malformations involving multiple body systems that may affect the timing but not the technical aspects of surgical correction of DA [2]. We describe surgical management strategy of DA in a newborn with massive hepatomegaly secondary to congenital TMD/AML.

## CASE REPORT

A female newborn with an antenatal diagnosis of DS on cell free DNA testing of maternal blood was delivered prematurely at 34 weeks gestation owing to polyhydramnios. Antenatal genetic testing had identified the DS to be due to an abnormal translocation of chromosome 21. No other congenital defect could be identified on maternal screening ultrasound studies. Massive hepatomegaly was identified after birth in addition to a pansystolic murmur over the precordium. The complete blood count (CBC) showed leukocyte count to be above 100,000/ mm3 with multiple immature cells of myeloid lineage. The neonate had non-bilious emesis on attempted feeding after birth; an upper gastro intestinal (GI) study showed anatomic distortion of the stomach and duodenum with a significantly delayed passage of contrast into the jejunum. A ventricular septal defect (VSD) at the junction of the membranous and muscular parts of the inter-ventricular septum was identified on echocardiography. The infant was started on Ara-C [Cytosine Arabinoside at a dose of 50mg/m2] for the treatment of TMD/AML on day 3 of life. A 5-day course completed the initial treatment regimen with significant resolution of the hepatomegaly after two weeks. The neonate was maintained on parenteral nutritive support in the interim.

A repeat upper GI study reconfirmed duodenal obstruction and an uneventful surgical correction was carried out sixteen days after birth. At operation, the liver had a normal consistency and was not unduly friable. There was a fenestrated duodenal web. The second and third parts of the duodenum were significantly stenosed and not amenable to a duodeno-duodenostomy, thus duodeno-jejunostomy was performed. The infant’s blood counts remained normal on follow up and no further chemotherapy was needed. Surgical correction of the VSD at 6 months of age is planned.

## DISCUSSION

TMD is seen in 10% of infants with DS [3]. While its morphological and phenotypic features are indistinguishable from AML all blasts in TMD carry trisomy 21 mutation. Diagnosis is based on the identification of two separate abnormal blood counts with >5% non-erythroid blast cells in infants less than three months of age with DS and any one of the following 5 clinical features: splenomegaly, hepatomegaly, lymphadenopathy, cardiac or pleural effusions [4]. Cases are categorized as low risk (no hepatomegaly or life threatening symptoms), intermediate risk (hepatomegaly with or without hepatic dysfunction), and high risk (life threatening symptoms, hepatic insufficiency). Spontaneous regression over a 3-7 month period has been reported in low risk cases [4]. Chemotherapy is indicated in infants with intermediate risk, and high risk TMD with life threatening symptoms such as hyperviscosity syndromes, blasts>10%, renal or hepatic insufficiency, respiratory insufficiency, congestive heart failure, disseminated intravascular coagulation (DIC) or hydrops. A mortality of 20% is seen in the latter presentations [6]. Cardiac malformations are seen in around 50% of cases of TMD and gastrointestinal abnormalities such as DA are seen in about 10% of afflicted infants [7]. Infants are at risk for subsequent development of AML (20-30%) and myeloproliferative disorders with the highest risk being in the first three years of life. A higher incidence of several pediatric surgical conditions have been noted in DS, in addition to DA, including esophageal atresia/tracheoesophageal fistula (0.3-0.5%), Hirschsprung’s disease (1-3%), anal stenosis and atresia (1-4%), and pyloric stenosis (0.3%) (8). However in DS with TMD/AML, only DA, tracheal stenosis and biliary atresia have been reported in the literature and there is scant information with regards to their management pertinent to the associated malignancy (4).

From a surgical perspective, the extreme hepatomegaly can complicate management of the duodenal atresia. The abnormal anatomy may confound interpretation of the upper GI study as the stomach is stretched and compressed by the large liver. Based on this experience, it is better to repeat the UGI study about ten days after chemotherapy course, as there is significant resolution of the hepatomegaly over that period with less anatomic distortion making interpretation of test results more straightforward. Once diagnosis is confirmed, timing of surgery in the 2-3 week window also allows better exposure of the atretic segment as the chemotherapy facilitates significant resolution of hepatomegaly and this helps to avoid retraction of a fragile liver infiltrated by leukemic blasts. Surgical timing in this time frame also avoids the neutropenic nadir that occurs anywhere from 3-4 weeks after chemotherapy. It is important to ensure that the platelet count and hemoglobin levels are adequate prior to the surgical intervention as the neonates are sensitive to the chemotherapy. These were not issues that we faced in our patient. Antibiotic prophylaxis is important and continuation of antibiotic coverage for 5-7 days after surgery is indicated because of an increased risk for S. epidermidis sepsis in TMD. 

To best of my knowledge this is the first report of an approach to the surgical management of DA in a neonate with DS and TMD/AML; which may help other surgeons to managing this complex problem.

## Footnotes

**Source of Support:** Nil

**Conflict of Interest:**None

